# Editorial: New horizons in stroke management, volume II

**DOI:** 10.3389/fnhum.2026.2023091

**Published:** 2026-10-23

**Authors:** William Huynh, Nasser Kashou

**Affiliations:** 1 Faculty of Medicine and Health, Randwick Clinical School, UNSW Sydney, Randwick, NSW, Australia; 2 Faculty of Medicine and Health, School of Medical Sciences, The University of Sydney, Sydney, NSW, Australia; 3 Kash Global Tech, Columbus, OH, United States

**Keywords:** cognitive screening, immunodepression, neuroinflammation, post-stroke depression, precision medicine, prognosis, robotic rehabilitation, stroke management

Stroke remains among the leading causes of death and acquired disability worldwide, and the therapeutic advances of recent decades have expanded the range of problems that contemporary stroke management must address. Reperfusion therapy has been extended to later time windows and to larger ischaemic cores, case fatality has fallen across many health systems, and a growing population of survivors consequently live for years with cognitive, affective and physical sequelae. The clinical problem has therefore shifted, at least in part, from improving survival toward characterizing the aftermath of acute stroke.

The first volume of this Research Topic addressed acute biomarkers, neuromodulation and rehabilitation technology. The present volume comprises 18 contributions, and its most striking feature is the breadth of disciplines from which they have emerged, appearing across eight Frontiers journals encompassing neurology, human neuroscience, psychiatry, neuroscience, surgery, pharmacology, endocrinology and nutrition; with the strongest submissions concerning inflammation, immune competence, metabolism, mood, cognition, surgical judgement and cost, frequently within the same patient.

Four contributions address the first hours and days after onset, each engaging a decision for which trial evidence remains incompletely defined.

Kaur et al. implemented an intravenous tirofiban protocol modeled on RESCUE BT2 at a United States comprehensive stroke center, comparing thirty-two treated patients with a historical standard-care cohort. No symptomatic or asymptomatic intracranial hemorrhage occurred by any of three standard definitions, and no thrombocytopenia was observed. Importantly, and in contrast to the trial on which the protocol was based, more than eighty per cent received tirofiban after early neurological deterioration had already occurred, at a median of 28.8 h from last known well. The authors therefore describe a rescue strategy for a population in whom current guidelines recommend no targeted therapy, and appropriately characterize their favorable neurological outcomes as hypothesis-generating.

Xie et al. analyzed 48,294 thrombolysed patients from the China Stroke Center Alliance, of whom 5,465 had a history of atrial fibrillation, thereby addressing a question on which previous studies have returned conflicting results. Following adjustment for age, sex, comorbidity, stroke severity and premorbid function, atrial fibrillation remained associated with in-hospital mortality and, with a larger effect, with cerebral hemorrhage.

Zhang et al. compared stereotactic aspiration of necrotic brain tissue, performed between 24 and 72 h after onset, with conservative management in 121 patients with malignant middle cerebral artery infarction. Of note, enrolment was restricted to patients aged 61 years and older, a group for whom the landmark decompressive hemicraniectomy trials provide limited guidance. Early cerebral herniation occurred in 8.5% of the aspiration group compared with 47.3%, and mortality in 19.1% compared with 64.9%, without significant difference in hemorrhage or intracranial infection.

Xie et al. contribute a health-economic dimension too infrequently addressed in acute stroke research, modeling edaravone dexborneol administered as a sublingual tablet vs. concentrated solution for injection. Using clinical inputs from the TASTE-SL and TASTE trials, the proportion achieving a modified Rankin Scale score of ≤ 1 did not differ significantly between formulations, while the oral formulation outperformed in cost-effectiveness. The more consequential implication may be one of accessibility, since an oral route removes the infusion infrastructure otherwise required.

Lin et al. prospectively sampled peripheral blood on days 1, 3 and 7 following endovascular therapy in acute anterior circulation large vessel occlusion, measuring monocyte human leukocyte antigen-DR (mHLA-DR) expression alongside interleukin-6, C-reactive protein and procalcitonin. Following multivariate adjustment, it was reduced mHLA-DR expression at day 1, together with dysphagia, that independently predicted stroke-associated pneumonia. As mHLA-DR is an established marker of monocyte activity and plays a central role in the immune response, this finding implicates stroke-induced immunodepression rather than excess inflammation. These findings complement the registry data of Xie et al. in which pneumonia affected more than a quarter of thrombolysed patients with atrial fibrillation. These immunological findings suggest both a possible mechanism and a way of identifying susceptible patients early.

Complications are not exclusively immune-mediated, however, and Zhang et al. address one that is largely iatrogenic and, in principle, preventable. In 305 patients with severe stroke, refeeding syndrome following initiation of enteral nutrition was accompanied by the characteristic fall in serum phosphorus and was associated with significantly higher rates of new acute liver injury, acute kidney injury, mechanical ventilation and 28-day mortality, together with worse modified Rankin Scale scores at discharge. The main implication is therefore one of prevention rather than prediction, and nutritional management in severe stroke may deserve more attention than it currently receives.

Six contributions address depression after stroke, the largest cluster in this Research Topic and, given that the condition affects approximately one third of survivors and remains under-recognized, this represents a much welcomed emphasis.

Four pursue a common strategy, namely the identification of at-risk patients using laboratory values obtained at admission. Su et al. prospectively followed 318 patients with first-ever acute ischaemic stroke, finding that those in the highest tertile of the systemic immune-inflammation index were 3.5 times more likely to develop depression at 3 months. In two complementary analyses of a single cohort at Shanxi Bethune Hospital, Kong et al. examined the admission uric acid to high-density lipoprotein cholesterol ratio and Kong et al. the non-high-density lipoprotein to high-density lipoprotein cholesterol ratio, reporting independent associations with 90-day depressive outcomes in both instances; in the latter, each unit increase conferred approximately 35% higher odds following adjustment. Luo et al. extended this approach to haemorrhagic stroke, demonstrating in 446 patients with spontaneous intracerebral hemorrhage that admission serum pre-albumin was lower among those subsequently diagnosed with depression. Given that pre-albumin is a negative acute-phase protein, the direction of this association is concordant with the inflammatory findings in the previous contributions discussed.

The practical appeal is worth noting, in that risk stratification may require no new assays but better use of measurements already present in the medical record. The predictive performance of any single index nonetheless remains modest, and the next step is to compare these markers directly and to test them prospectively, for which these studies provide a useful foundation.

Two reviews supply the mechanistic account that renders such associations biologically plausible. Tang et al. review impaired hippocampal neurogenesis as a mechanism of post-stroke depression. Ischaemic injury does increase neural stem cell proliferation acutely, yet the hippocampus derives little benefit: proliferation is short-lived, few progenitors survive, cell fate shifts toward astrogenesis, and the neurons produced are often abnormal in shape and location. The authors also review hypothalamic-pituitary-adrenal axis hyperactivity, neuroinflammation, reduced brain-derived neurotrophic factor signaling and monoaminergic disturbance. One pathway is of particular interest here: interleukin-6 drives indoleamine 2,3-dioxygenase, which diverts tryptophan away from serotonin synthesis. This is precisely the biology that would link a raised inflammatory index at admission to depression months later.

Li and Gao review depression following intracerebral hemorrhage, reported between 18 and 60% and less well characterized than its ischaemic counterpart. Alongside monoamine imbalance and bidirectional neuroinflammation, they emphasize oxidative stress and iron dysregulation arising from the breakdown of extravasated blood, mechanisms that are specific to hemorrhage and that argue against simply applying frameworks developed for ischaemic stroke.

Tanglay et al. compared the Addenbrooke's Cognitive Examination-III against the stroke-specific Oxford Cognitive Screen in a small cohort of patients assessed within 7 days of first-ever stroke. Although the ACE-III demonstrated good discriminatory capacity at the group level, neither the standard or stroke specific cut-off satisfied established minimum accuracy criteria for cognitive screening after stroke; five patients identified by the OCS were missed, predominantly owing to attentional deficits, while two aphasic but cognitively intact patients were misclassified as impaired. Impairments in praxis and calculation were those associated with functional dependence, and both are underrepresented in general cognitive instruments.

Lu et al. recorded eye movements during free visual exploration of familiar dramatic character faces in 16 patients and twenty-three controls. Prolonged fixation duration, restricted saccadic movement and reduced scanpath length distinguished the stroke group, and a machine learning classifier integrating these features achieved an accuracy of 87.18%. The particular merit of the paradigm lies in its independence from instruction-following, which may render it feasible in patients unable to comply with structured assessment.

Hamadi et al. applied targeted whole-exome sequencing in a Saudi cohort from Tabuk, identifying eleven novel variants in *GSTT4* alongside variants in cholesterol-associated genes and common polymorphisms in *MTHFR, KLF14, NOS3* and *ACE*. Given that most stroke genetics derives from populations of European ancestry, adequately characterized non-European cohorts are of considerable value.

Liu et al. analyzed 1,620 patients with acute ischaemic stroke and hypertension, and while elevated TyG-BMI was associated with one-year recurrence overall, the association was confined almost entirely to women with no evidence of association among men. The sex-specific pattern is the arguably the more notable finding here.

Ye et al. applied latent class mixed modeling to serial modified Rankin Scale measurements in 127 patients with cerebral venous sinus thrombosis, demonstrating that long-term recovery is better described by two distinct trajectories than by a continuum. Together with younger age, a higher venous collateral circulation score was strongly protective against belonging to the poor-recovery class, thereby identifying a structural and imageable determinant of outcome.

Amirbekova et al. report a randomized controlled trial of the ReHand portable soft exoskeleton in 120 patients during the subacute phase, delivered five times weekly over 8 weeks in addition to standard therapy. The minimal clinically important difference of 5.25 points on the Fugl-Meyer Assessment for the Upper Extremity was achieved by 91.7% of the intervention group compared with 43.3% of controls, with corresponding gains reflected in the Barthel Index and Functional Independence Measure. Given that upper limb recovery remains one of the most difficult problems in stroke rehabilitation, and that the subacute window represents the period of greatest neuroplastic capacity, trials of this kind therefore address an important gap.

Taken together, these 18 contributions describe a field in which progress is occurring across several distinct areas. The most consistent theme across the collection is the effort to extract prognostic information from data that already exist, whether admission blood results, registry records or routine bedside assessment. Such approaches are widely applicable, and are of particular relevance to centers without access to advanced imaging or specialized biomarker panels. Low-cost prognostic information is essential if risk stratification is to be feasible at scale, and several studies collected here represent progress toward that objective.

